# Isochlorogenic Acid C Mitigates CCl_4_-Induced Kidney Ferroptosis, Inflammation, and Fibrogenesis in Mice: Involvement of the SESN2/MAPKs and Hippo Pathways

**DOI:** 10.3390/antiox15101349

**Published:** 2026-10-19

**Authors:** Jieqiong Ma, Yuhan Jiang, Ming Zhou, Ruiqi Li, Chanmin Liu

**Affiliations:** 1 School of Medicine, Sichuan University of Science and Engineering, Xueyuan Road, Zigong 643000, China; 15265205198@163.com (Y.J.); mzhou@suse.edu.cn (M.Z.); liruiqi94@163.com (R.L.); 2 School of Life Science, Jiangsu Normal University, No. 101, Shanghai Road, Tangshan New Area, Xuzhou 221116, China

**Keywords:** isochlorogenic acid C, renal fibrosis, oxidative stress, inflammation, ferroptosis, SESN2

## Abstract

Renal damage is closely related to oxidative stress, inflammation, ferroptosis, and fibrosis. Renal fibrosis is closely related to oxidative stress, inflammation, and ferroptosis. Isochlorogenic acid C (IaC) has been demonstrated to have a protective effect against kidney injury in both in vivo and in vitro studies. Nonetheless, it remains uncertain whether IaC can mitigate kidney dysfunction induced by carbon tetrachloride (CCl4). In this study, we examined the protective effects of IaC on renal inflammation, ferroptosis, and fibrosis, as well as the underlying regulatory mechanisms. An experimental model of kidney injury was developed using ICR mice (in vivo) and HK-2 cells (in vitro), which were treated with CCl4 or TGFβ1. Gene/protein expressions were detected using RT-qPCR, Western blotting, and immunohistochemistry assays. In addition, to identify the potential mechanisms involved in this process, we conducted RNA interference and signaling pathway inhibition analysis. IaC alleviated CCl4-induced renal dysfunction and fibrosis. It resulted in the down-regulation of fibrosis-associated markers, specifically collagen I and α-SMA. Furthermore, IaC mitigated CCl4-induced renal inflammation by reducing the activities of TNF-α, IL-6, and NF-κB p65. IaC led to an up-regulation in the activities of GSH, SOD, and GPx in the renal tissues of mice. IaC effectively inhibited the elevation of renal ferroptosis-associated markers induced by CCl4, as evidenced by the reduced Fe^2+^ levels and the increased expression of GSH, GPX4, and SLC7A11. Moreover, IaC significantly enhanced the activation of Sestrin2 (SESN2). IaC resulted in a down-regulation of the expression levels of TGFβ1, YAP, TAZ, p-ERK, p-JNK, and p-p38 in the kidneys. Furthermore, IaC demonstrated an inhibitory effect on inflammation and fibrosis within an in vitro model of TGFβ-induced fibrosis. However, treatment with the inhibitors of MAPKs and YAP significantly enhanced the effects of IaC in TGFβ-stimulated HK-2 cells. Sestrin2 silencing also reversed the effects of IaC in TGFβ-stimulated HK-2 cells. IaC demonstrated effective docking with the active sites of the target proteins SESN2, GPX4, and YAP, thereby corroborating the predicted compatibility of interactions between IaC and these proteins. Our study demonstrates that IaC ameliorates CCl4-induced renal inflammation and fibrosis through modulation of the Hippo pathway and the SESN2/MAPKs pathways. IaC is a promising natural compound that can be used to treat kidney damage.

## 1. Introduction

Kidney damage results in abnormal function and various diseases, with fibrosis being the end stage of most chronic kidney conditions. This fibrosis is marked by excessive extracellular matrix buildup, including cross-linked collagen, causing tissue stiffening and organ dysfunction [1,2,3]. Sestrin2 (SESN2) is a stress-inducible protein that helps maintain redox balance by regulating metabolism and is increased during various stress conditions. It also reduces septic intestinal inflammation by inhibiting ferroptosis and the MAPK pathway [4,5]. SESN2 knockdown markedly suppressed proliferation, migration, invasion, and apoptosis of OSCC cells through the MAPK pathway [6]. Knockout of SESN2 reduces glomerulonephritis and fibrosis through the Nrf2/HO-1 signaling pathway [7]. SESN2 inhibited pulmonary fibrosis, inflammation, and ferroptosis by the Nrf2 pathway [8,9]. The Sestrin2 signaling pathway is engaged in the protection of diabetic nephropathy and renal fibrosis [10]. The Hippo pathway is a key evolutionary signaling cascade involved in cell growth, organ development, tissue repair, and immune response. While it mainly regulates YAP/TAZ activity, YAP/TAZ are also influenced by various mechanical signals [11,12,13,14]. Furthermore, SESN2 overexpression inhibited oxidative stress, proliferation, and fibronectin by suppressing Hippo pathway in diabetic nephropathy [15]. Stimulation of SESN2 ameliorates hepatic fibrogenesis induced by carbon tetrachloride (CCl4) [16,17]. CCl4 is known to induce inflammation and fibrosis across various animal tissues by up-regulating the expression of α-smooth muscle actin (α-SMA), TGF-β, NF-κB, TNF-α, and IL-6 [12,18]. Moreover, CCl4-induced liver fibrosis reduces enzymatic antioxidants, raises reactive oxygen species levels, and increases fibrogenic factors like YAP and TAZ, showing the link between these pathways in fibrogenesis [18]. Inhibiting YAP activity can alleviate CCl4-induced liver fibrosis by blocking the JNK/YAP pathway, reducing oxidative stress and fibrogenic responses, highlighting its potential in therapy [19,20]. Previous studies have shown that CCl4 causes kidney inflammation and fibrosis in rodents. Our initial research found that CCl4 triggers oxidative stress, inflammation, apoptosis, and fibrosis in mouse kidneys via the MAPK pathway [3,21,22]. However, the precise mechanisms underlying CCl4-induced kidney fibrosis remain to be elucidated.

Isochlorogenic acid C (IaC), a di-O-caffeoyl derivative of chlorogenic acid, is a prominent extract derived from various herbal plants, including *Lonicera japonica*, *Hyssopus cuspidatus*, *Chrysanthemum morifolium*, and *Crassocephalum crepidioides*. This compound has been utilized in the production of scented teas, medicinal teas, and soft drinks [23,24,25]. Numerous studies have reported that IaC exerts several pharmacological properties, including anti-oxidative, anti-hyperlipidemic, anti-inflammatory, antiviral and anticancer activities [23,24,25]. However, whether IaC can improve renal inflammation and fibrosis remains unknown.

Based on the pre-laboratory research, we hypothesized that IaC has the positive effects on CCl4-induced renal ferroptosis, inflammation and fibrosis. The effects of IaC on CCl4-induced renal ferroptosis, inflammation, and fibrosis and the underlying mechanism were investigated in this study. We further confirmed that its positive role was mediated by activating antioxidant enzymes and inhibiting the SESN2/MAPKs and Hippo signaling pathways.

## 2. Methods

### 2.1. Chemicals and Reagents

Isochlorogenic acid C (CAS No. 57378-72-0, purity verified by HPLC ≥ 98%) was procured from Chengdu Preferred Biological Technology Co., Ltd. (Chengdu, China). The YAP inhibitor (20 μM, Verteporfin (VP)), JNK inhibitor (20 μM, SP600125(SP)), p38 inhibitor (20 μM, SB203580(SB)), ERK inhibitor (20 μM, PD98059(PD)), and CCl4 were purchased from Sigma (St. Louis, MO, USA). Antibodies SLC7A11 (26864-1-AP), GAPDH (60004-1-Ig), and SESN2 (66297-1-Ig) were obtained from Proteintech BioTechnology (Wuhan, China). Antibodies IL-6 (TD6087), TNF-α (PY19810) were obtained from Abmart BioTechnology (Shanghai, China). Antibodies GPx4 (ab125066), p-JUN1/2(ab124956), JNK1/2(ab179461), p-ERK1/2(ab214036), ERK1/2 (ab184699), p-p38 (ab195049), p38 (ab170099) were supplied by Abcam (Shanghai, China).

### 2.2. Animal Experiment Design and Ethics

Fifty male 8-week-old ICR mice were provided from Beijing HFK Bioscience Co., Ltd., (Beijing, China). All experimental animal procedures were approved by the Animal Experiment Ethics Committee of Jiangsu Normal University (approval No. JSNU-IACUC-2024065). Mice were housed in a room maintained under the following conditions: a temperature of 22 ± 3 °C, relative humidity of 55 ± 5%, and a 12 h light-dark cycle. During the experiments, all mice had free access to food and water. The CCl4 solution 2 mL/kg was injected intraperitoneally twice per week for 4 weeks. The IaC dissolved in normal saline and was administrated daily by gavage in the treatment groups. After one week of adaptive rearing, the mice were randomly divided into five groups (10 mice/group): (1) the normal control group; (2) CCl4 group; (3) CCl4 + IaC (10 mg/kg) group; (4) CCl4 + IaC (20 mg/kg) group: (5) IaC (20 mg/kg) control group. The control group (1) received intraperitoneal injections of 2 mL/kg olive oil twice a week, while 2 mL/kg physiological saline was administered orally daily. In groups (2), (3) and (4), the procedure of CCl4-induced renal fibrosis was performed as described [1,2,3]. In groups (3), (4) and (5), mice were also supplied with IaC 10 or 20 mg/kg, intragastrically once daily [24]. Three mice were randomly selected for tissue analysis, while the others were used for biochemical and molecular analysis.

At the end of 4 weeks, mice were sacrificed by decapitation. Blood and kidneys were collected immediately for future experiments.

### 2.3. Cell Culture and Treatment

In this study, human proximal tubular epithelial cells (HK-2) (C1116; Shanghai Yingwan Biotechnology Co., Ltd., Shanghai, China) were utilized. The HK-2 cells were maintained in RPMI-1640 medium (Gibco, Waltham, MA, USA) supplemented with 1% penicillin/streptomycin, at 37 °C in a humidified atmosphere containing 5% CO_2_. The experimental findings indicated that ICACs at concentrations of 100 μM or lower did not impact cell viability, nor did they demonstrate significant cytotoxic effects [25]. Human kidney cells were seeded in 96-well plates at a density of 5 × 10^3^ cells/well. After serum starvation for 24 h, cells were pretreated with IaC (50 μM) for 12 h, incubated with 10 ng/mL TGF-β1 (Sigma-Aldrich, Beijing, China) treatment for another 24 h. All the inhibitors at the indicated doses were used to pretreat the cells 1 h prior to IaC treatment. Then cells were harvested and subjected to future experiments. The usage method and dosage selection of inhibitors refer to previous experimental studies [16,25]. The SESN2 knockdown model (siS) was achieved using small interfering RNA (siRNA) according to the manufacturer’s protocol (RiboBio, Guangzhou, China) [26]. The sequences of Sestrin2 siRNA (stB0013970, RiboBio) included Sestrin2-1 (CACCCTGACTACTTTACCA), Sestrin2-2 (CCCGAAGAATGTACAACCT), and Sestrin2-3 (CCTATAGCCTCACCTAC AA). Transfection was carried out using the Ribo FECT CP Transfection Kit (C10511-05, RiboBio). Sestrin2-2 siRNA was employed for subsequent experiments following confirmation of gene knockdown efficiency via Western blot analysis. HK-2 cells were transfected with 100 nM Sestrin2 siRNA or negative control siRNA (Si-Con) for 24 h, following the manufacturer’s protocol [26].

### 2.4. Biochemical Analysis

The contents of creatinine (#C011-2-1), urea (#C013-1-1), uric acid (#C012-1-1), malondialdehyde (MDA,# A003-1-2), glutathione (GSH, #A006-1-1), and the activities of superoxide dismutase (SOD,# A001-3-2), and glutathione peroxidase (GPx,# A005-1-2) in serum and kidneys were analyzed by using commercial kits from Jiancheng Institute of Biotechnology (Nanjing, China) [21]. The level of ferrous iron was also detected using an iron assay kit according to the protocol of the manufacturer (ab83366, Abcam, Cambridge, MA, USA). A total of 5 μL of assay buffer was added to measure Fe^2+^ levels according to the manufacturer’s protocol. Absorbance was measured at 593 nm by a microplate reader [9].

### 2.5. Histology and Immunohistochemistry (IHC)

Kidney tissues were obtained to be fixed in 4% paraformaldehyde, followed by embedding in paraffin, cutting into 5 μm sections, and staining with hematoxylin and eosin (H&E) kits (#D006-1-1) and Masson stain kits (#D026-1-1) (Jiancheng Institute of Biotechnology, Nanjing, China). Kidney damage and fibrosis was quantified using ImageJ 1.42 software (NIH, Bethesda, MD, USA). Damage was graded on a scale of 0–4 (0 = normal; 0.5 = small focal areas; 1 = involvement of less than 10% of the cortices and outer medullae; 2 = 10–25% involvement of the cortices and outer medullae; 3 = 25–75% involvement of the cortices and outer medullae; 4 = extensive damage involving more than 75% of the cortices and outer medullae). We also evaluated the number of tubules with cellular necrosis from the renal cortices and outer medulla by grid fields (0.245 mm^2^). Glomerular lesions (total score: 0–12) included glomerular hypercellularity, glomerular segmental lesions (crescents, adhesions and segmental sclerosis) and global glomerular sclerosis. The glomerular lesion score of each type was determined as follows: 0, no lesion; 1, lesion in <10% of glomeruli; 2, lesion in >10% but <25% of glomeruli; 3, lesion in >25% and <50% of glomeruli; and 4, lesion in >50% of glomeruli. Five different visual fields were chosen from each section at random to calculate kidney injury markers [21].

For immunohistochemistry assays, the kidney sections were processed according to the instructions of the KeyGen Biotech One-Step IHC Assay kit (Nanjing, China) [7]. In brief, the sections were incubated with SESN2, GPX4, and YAP antibodies at a dilution of 1:200, followed by incubation with the secondary antibody. Five different visual fields were chosen from each section at random to calculate the mean optical density (OD). Images were analyzed using the ImageJ software.

### 2.6. RT-qPCR Analysis

RT-qPCR analysis was performed following previous studies [24]. Briefly, total RNA was extracted using TRI pure reagent according to the manufacturer’s instructions. Then cDNA synthesis and amplification was performed using the commercial kits (Accurate Biology, Changsha China). First-strand cDNA was synthesized using the EntiLink™ 1st Strand cDNA Synthesis Super Mix (ELK Biotechnology, Wuhan, China). Real-time PCR was performed using the EnTurbo™ SYBR Green PCR SuperMix (ELK Biotechnology, Wuhan, China) on a QuantStudio 6 Flex System. The cDNA samples of each gene were amplified 3 times in parallel, and the mean value of Ct was used for analysis. The PCR conditions were as follows: 95 °C for 30 s, followed by 40 cycles of 95 °C for 10 s, 58 °C for 30 s and 72 °C for 30 s. Relative expression levels were calculated using the 2^−^ΔΔCT method with β-actin as the internal control. Gene expression of TNF-α, IL-6, α-SMA, COL1A1, SLC7A11, GPX4, SESN2, TGF-β1, YAP, TAZ, and β-actin was quantified using RT-PCR Thermal Cycler (Analytik Jena, Jena, Germany). The primers sequences used are shown in Table 1.

### 2.7. Western Blot Analysis

The protein expression levels were analyzed by Western blot according to the manufacturer’s guidelines (Bio/Rad, Hercules, CA, USA). The protein was extracted from kidney tissues using commercial kits (P0013B, Beyotime Biotechnology, Shanghai, China). The protein samples were collected after centrifugation at 12,000 rpm for 10 min at 4 °C, and the protein concentrations were measured using a bicinchoninic acid (BCA) protein assay kit (#23225, Thermo, Rockford, IL, USA). An equal amount of protein was loaded and separated on 8~15% sodium dodecyl sulfate-polyacrylamide gels and transferred onto polyvinylidene difluoride membranes (PVDF, Merck Millipore Ltd., IPVH00010, Darmstadt, Germany). The transferred membranes were blocked with QuickBlockTM solution (P0252, Beyotime Biotechnology, Shanghai, China) for 15 min at room temperature, washed in PBST (0.1% Tween-20 in PBS), and incubated overnight with the primary antibodies (1:5000), followed by development with secondary antibodies (1:5000) for 1 h at room temperature. The blots were quantified using Image J 1.42 software (NIH, Bethesda, MD, USA) [21].

### 2.8. Molecular Docking

To analyze the binding affinities and modes of interaction between IaC and their targets, Autodock Vina 1.2.3 was employed with IaC as the ligand and proteins SESN2, GPX4, and YAP as the receptors. The 3D structures of IAC were retrieved from PubChem Compound (https://pubchem.ncbi.nlm.nih.gov/, accessed on 20 December 2025). The 3D coordinates of SESN2 (PDB ID, 6N0M; resolution, 3.30 Å), GPX4 (PDB ID, 9RF1; resolution, 1.90 Å), and YAP (PDB ID, 6G6X; resolution, 1.13 Å) were downloaded from the PDB (http://www.rcsb.org/). Molecular docking studies were performed by Autodock Vina 1.2.3.

### 2.9. Statistical Analysis

Statistical analysis was conducted with GraphPad Prism 8.0. Statistical significances were determined using one-way ANOVA and post hoc Tukey test. All results were shown as mean ± SEM. *p* < 0.05 was considered as significant value.

## 3. Results

### 3.1. IaC Reversed Renal Dysfunction

**Figure 1:**
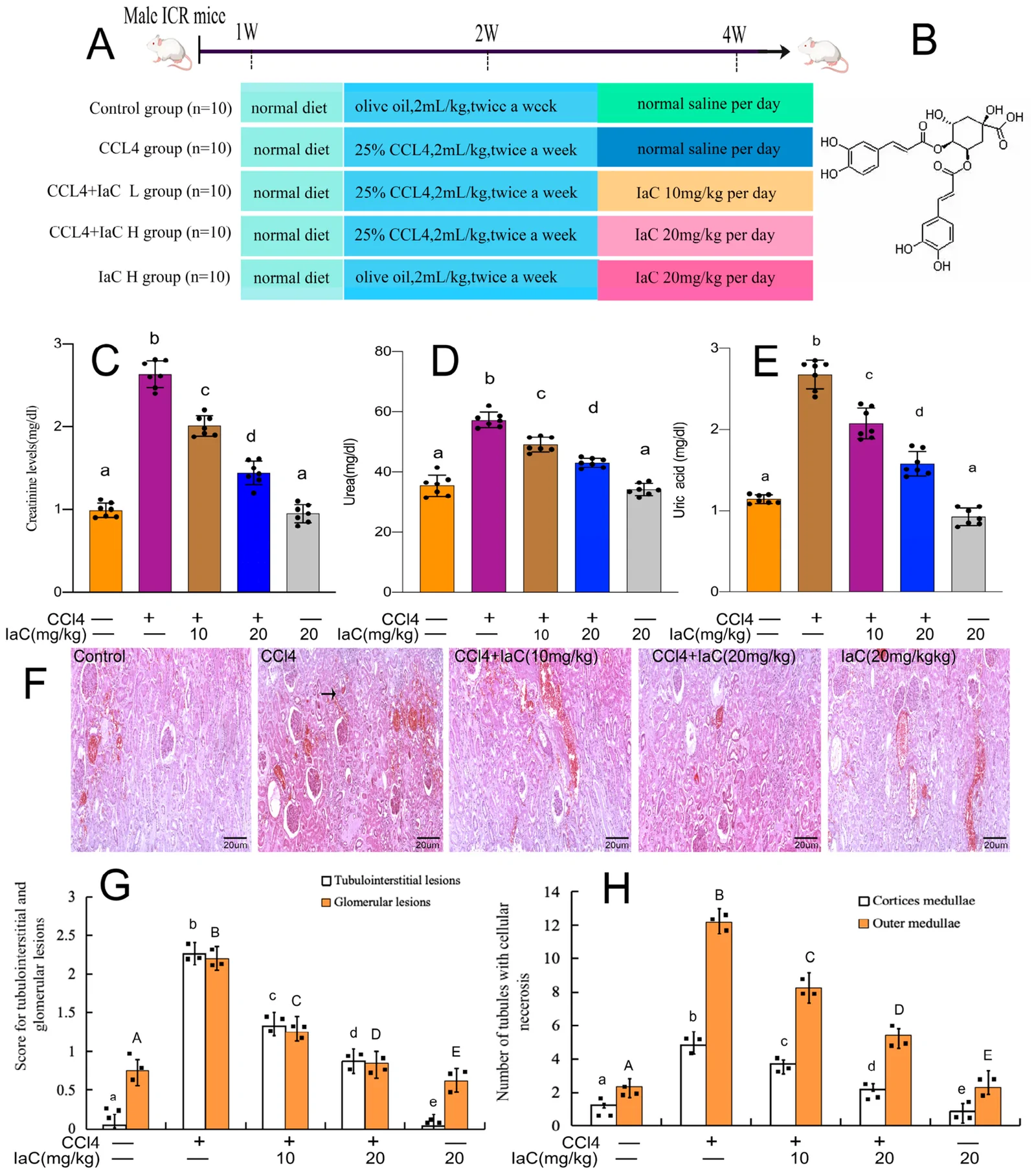
Isochlorogenic acid C (IaC) improved dysfunction and histological alterations in the kidneys. (**A**) The experimental design and grouping of mice; (**B**) the chemical structure of IaC; (**C**) creatinine levels; (**D**) urea levels; (**E**) uric acid levels; (**F**) representative histological images of hematoxylin and eosin (H&E) in kidney sections (×200); (**G**) the score for tubulointerstitial and glomerular lesions; (**H**) the number of tubules with cellular necrosis. The black arrow indicates infiltrating leukocytes. Data are expressed as mean ± S.E.M. (*n* = 7 for kidney function; n = 3 for kidney damage markers). Different letters placed above columns represent significant differences between treatments (*p* < 0.05).

To confirm the renoprotective effects of IaC on inflammation and fibrosis, a mouse model of CCl4-induced kidney damage was established. When compared with the control, CCl4 treatment increased levels of serum creatinine, urea, and uric acid by 154.29%, 94.06%, and 153.21%, respectively. The treatment with IaC (10 and 20 mg/kg) decreased these indicators compared to the CCl4 group, suggesting a reversal of the CCl4-induced damage effects (Figure 1A–E).

### 3.2. IaC Alleviated Renal Histopathological Alterations

The histopathological changes in mouse kidneys were detected by HE staining. As depicted in Figure 1, compared with the control, the CCl4 group exhibited kidney damage, including degenerated and necrotic renal tubules, shrunken glomerular tufts, desquamated cell nuclei, necrosis, exfoliation, and coalescing inflammatory aggregation. However, the renal damage, glomerular injury, and inflammatory responses were significantly alleviated by IaC supplementation. Moreover, CCl4 treatment increased the score for tubulointerstitial and glomerular lesions. After IaC supplementation, the histopathological score decreased, indicating its positive influence on the histological property of the kidney structure (Figure 1F–H).

### 3.3. IaC Alleviated Renal Fibrosis Alterations

**Figure 2:**
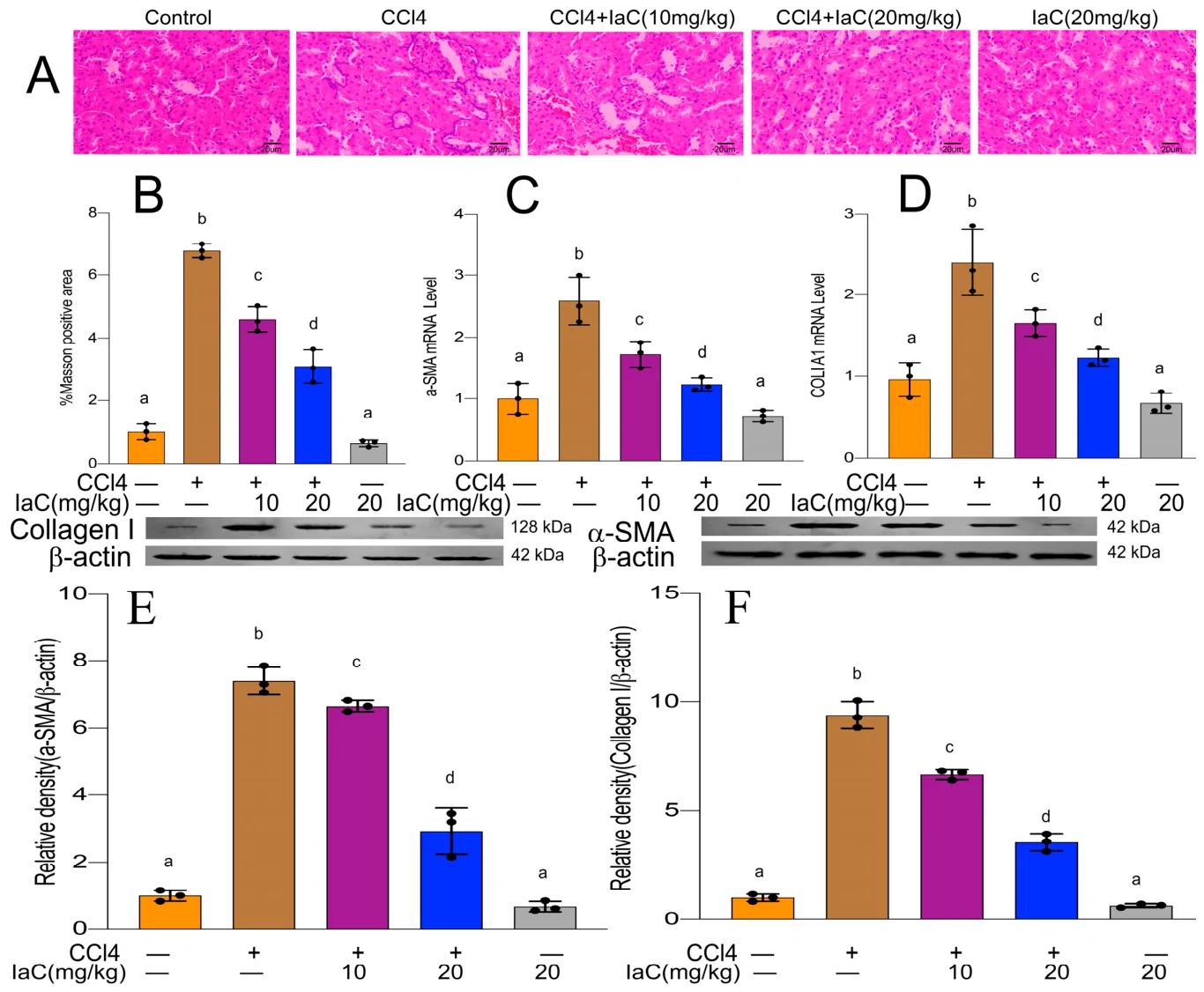
Isochlorogenic acid C (IaC) inhibited CCl4-induced fibrosis in the kidneys. (**A**) Representative histological images of fibrosis in kidney sections (×200); (**B**) relative area of Masson’s trichrome staining; (**C**) *α-SMA* mRNA levels; (**D**) *COL1A1* mRNA levels; (**E**) relative density analysis of the α-SMA protein bands; (**F**) relative density analysis of the collagen I protein bands. The vehicle control is set as 1.0. Data are expressed as mean ± S.E.M. (*n* = 3 for histological/molecular analyses). Different letters placed above columns represent significant differences between treatments (*p* < 0.05).

Kidney fibrosis was further determined. Masson’s trichrome staining showed that a strongly blue-stained area was observed in renal tissue, indicating renal fibrosis. Compared with the control group, the mRNA expression of *α-SMA* and *COL1A1* in the CCl4 group significantly increased (141.73% and 101.56%). Meanwhile, Western blotting results indicate that the CCl4-challenged mice exhibited significant characteristics of renal fibrosis, including increased expression levels of α-SMA and collagen I when compared with the control (Figure 2). However, IaC supplementation reversed renal fibrosis and the up-regulated expression of α-SMA and collagen I, demonstrating the protective role of IaC in renal fibrosis.

### 3.4. IaC Rescued Oxidative Stress in Kidneys

Oxidative damage is an important contributor to kidney damage. Therefore, the key indices related to oxidative damage (MDA, SOD, GPx, and GSH) were determined. IaC treatment hampered renal oxidative stress in the CCl4-challenged mice. CCl4 increased MDA content by 58.07% and reduced SOD, GPx, and GSH activities by 43.55%, 26.59%, and 49.53%, respectively. However, IaC supplementation reduced these indicators compared to the CCl4 group (Table 2).

### 3.5. IaC Inhibited the Elevation of Renal Ferroptosis-Associated Markers Induced by CCl4

**Figure 3:**
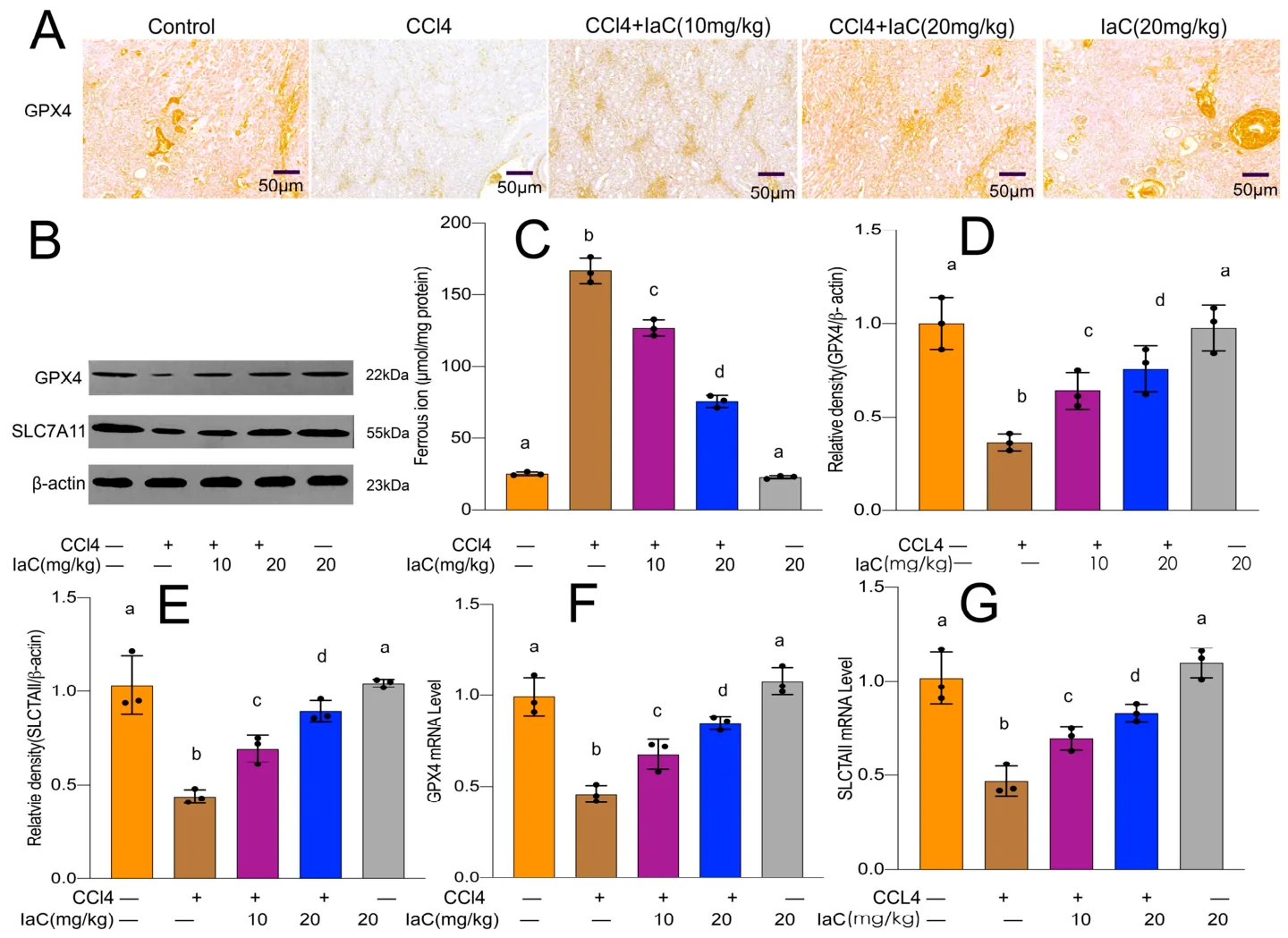
Isochlorogenic acid C (IaC) inhibited the elevation of renal ferroptosis-associated markers induced by CCl4. (**A**) Expression of GPX4 in kidney through immunohistochemistry, ×200. (**B**) GPX4 and SLC7A11 detected by Western blot; (**C**) Fe^2+^ levels; (**D**) the quantitative analysis of the GPX4 protein bands; (**E**) the quantitative analysis of the SLC7A11 protein bands; (**F**) *GPX4* mRNA levels; (**G**) *SLC7A11* mRNA levels. The vehicle control is set as 1.0. Data are expressed as mean ± S.E.M. (*n* = 3 for histological/molecular analyses). Different letters placed above columns represent significant differences between treatments (*p* < 0.05).

We further investigated ferroptosis, which is one interconnected pathway through which oxidative stress is linked to kidney damage. As shown in Figure 3, the level of Fe^2+^ significantly increased in kidney tissues following CCl4 treatment. The mRNA expression levels of *GPX4* and *SLC7A11* in the CCl4 group were all reduced compared with the control group. Pretreatment with IaC partially ameliorated the alterations in ferrous levels and ferroptosis-associated markers induced by CCl4, as depicted in Figure 3. Subsequently, immunohistochemical techniques were utilized to evaluate the protein expression of GPX4. In renal tissue, the administration of CCl4 significantly diminished the staining intensity of GPX4 expression. In contrast, treatment with IaC led to an increased staining intensity of GPX4 expression. These findings, in conjunction with the alterations in MDA and GSH levels presented in Table 2, suggest that IaC attenuates ferroptosis-associated markers in renal tissue.

### 3.6. IaC Suppressed Renal Inflammation

**Figure 4:**
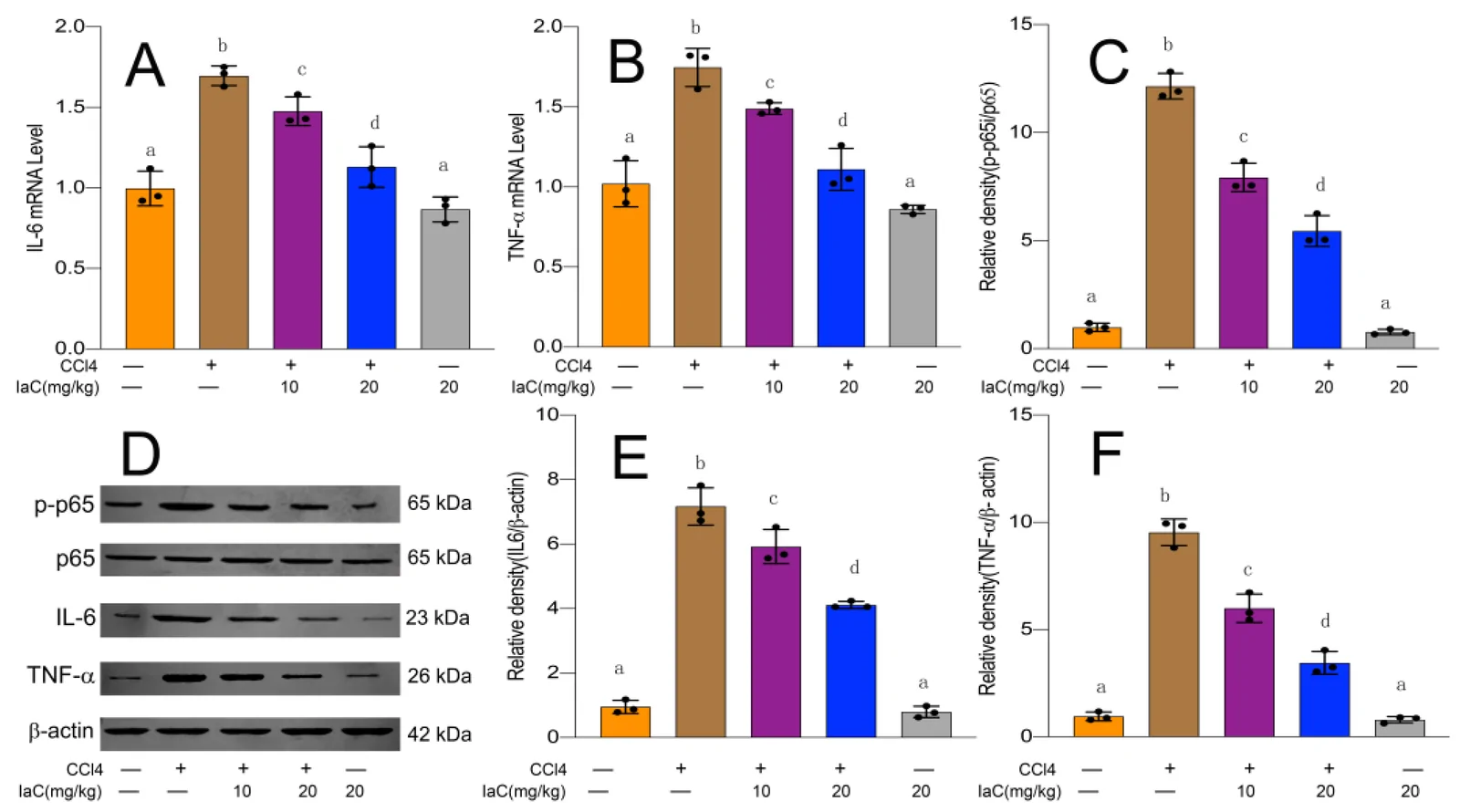
Isochlorogenic acid C (IaC) inhibited inflammation in the kidneys of mice. (**A**) *IL-6* mRNA levels; (**B**) *TNF-α* mRNA levels; (**C**) the quantitative analysis of the p-p65 protein bands; (**D**) IL-6, TNF-α, and p-p65 detected by Western blot; (**E**) the quantitative analysis of the IL-6 protein bands; (**F**) the quantitative analysis of the TNF-α protein bands. The vehicle control is set as 1.0. Data are expressed as mean ± S.E.M. (*n* = 3 for histological/molecular analyses). Different letters placed above columns represent significant differences between treatments (*p* < 0.05).

Studies have shown that the CCl4 stimulation caused the inflammatory response, promoting the synthesis and secretion of inflammatory factors. We found that CCl4 significantly up-regulated the mRNA levels of *TNF-α* and *IL-6* in renal tissue (Figure 4). We also used Western blot to evaluate the protein expression of TNF-α, IL-6, and NF-kB p65 phosphorylation in renal tissue. As displayed in Figure 4, in contrast to the control, TNF-α, IL-6, and NF-kB p65 phosphorylation increased after CCl4 stimulation. IaC supplementation decreased the activation of those inflammatory factors (*p* < 0.05).

### 3.7. IaC Inhibited the SESN2/MAPKs Pathway

**Figure 5:**
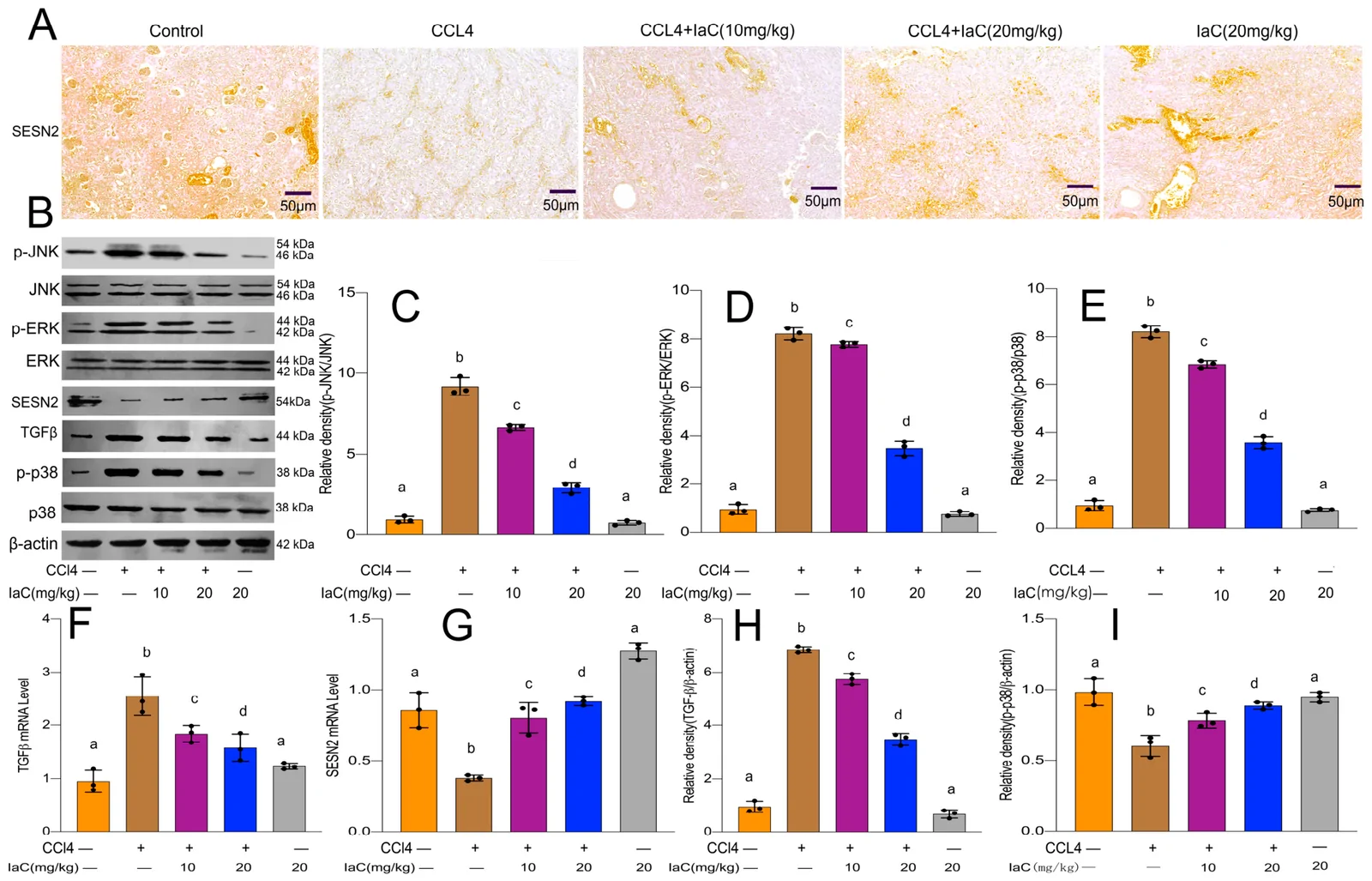
Isochlorogenic acid C (IaC) modulated the SESN2/MAPKs pathways in the kidneys. (**A**) Expression of SESN2 in kidney through immunohistochemistry, ×200. (**B**) SESN2, TGFβ1, JNK1/2, ERK1/2, and p38 detected by Western blot; (**C**) the quantitative analysis of the p-JNK1/2 protein bands; (**D**) the quantitative analysis of the p-ERK1/2 protein bands; (**E**) the quantitative analysis of the p-p38 protein bands; (**F**) *TGFβ1* mRNA levels; (**G**) *SESN2* mRNA levels; (**H**) the quantitative analysis of the TGFβ1 protein bands; (**I**) the quantitative analysis of the SESN2 protein bands. JNK1/2, ERK1/2, p38, and β-actin were probed as the internal control in relative density analysis. The vehicle control is set as 1.0. Data are expressed as mean ± S.E.M. (*n* = 3 for histological/molecular analyses). Different letters placed above columns represent significant differences between treatments (*p* < 0.05).

To explore the potential mechanism of IaC in modulating renal fibrosis, the SESN2/MAPKs pathway was examined (Figure 5). Western blotting assays illustrate that the CCl4 group had a lower expression level of SESN2 than the control, while the administration of IaC significantly elevated SESN2. Immunohistochemical analysis revealed a substantial expression of SESN2 in the renal tissues of control mice. Conversely, administration of CCl4 resulted in a marked reduction in SESN2 protein expression levels. Notably, subsequent treatment with IaC led to a significant up-regulation of SESN2 protein expression relative to the CCl4-treated group. In addition, we found that CCl4 up-regulated the mRNA levels of *TGFβ1* in renal tissue, and IaC treatment significantly down-regulated the mRNA levels of these genes. Western blotting assays showed that the level of TGFβ in the CCl4 group exhibited a notable increase, and IaC treatment decreased the TGFβ level compared with that in the CCl4 group. Moreover, in the CCl4 group, p-p38, p-JNK1/2, and p-ERK1/2 were up-regulated (Figure 5C–E). Contrary to the CCl4 group, the administration of IaC obviously reduced phosphorylation levels of these proteins dose-dependently.

### 3.8. IaC Inhibited YAP/TAZ Signaling Pathway in Kidneys

**Figure 6:**
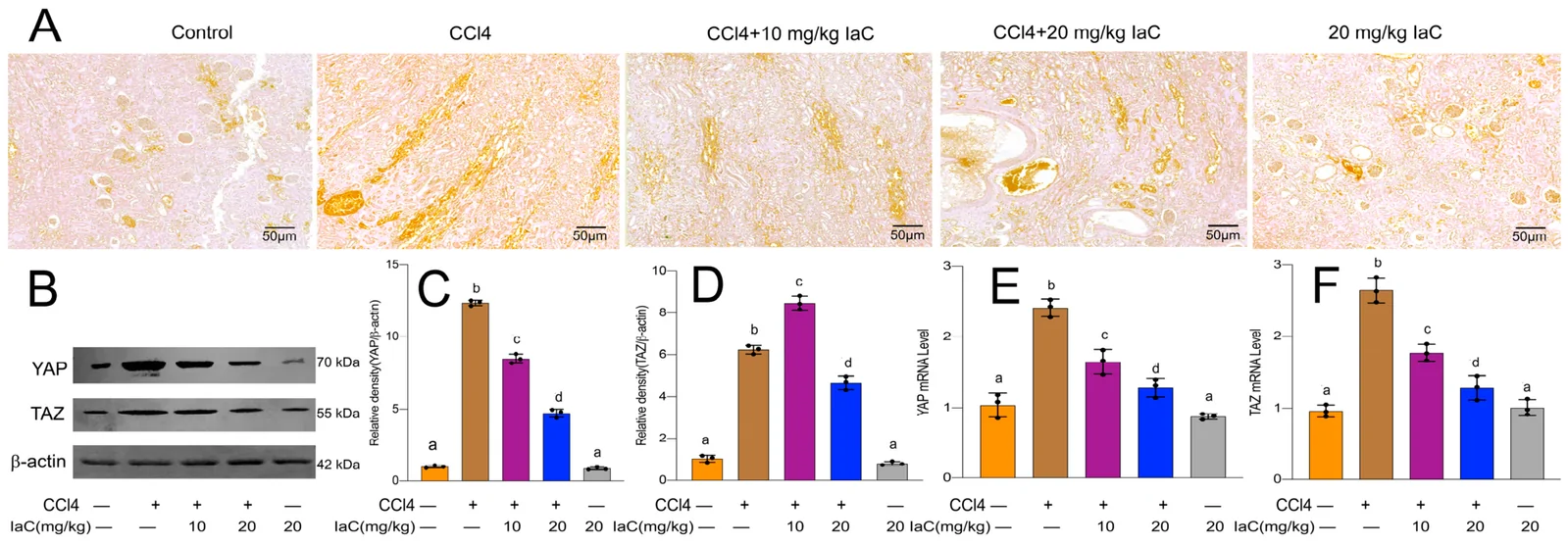
Isochlorogenic acid C (IaC) modulated the YAP/TAZ pathways in the kidneys. (**A**) Expression of YAP in kidneys through immunohistochemistry, ×200. (**B**) YAP and TAZ detected by Western blot; (**C**) the quantitative analysis of the YAP protein bands; (**D**) the quantitative analysis of the TAZ protein bands; (**E**) *YAP* mRNA levels; (**F**) *TAZ* mRNA levels. β-actin was probed as the internal control in relative density analysis. The vehicle control is set as 1.0. Data are expressed as mean ± S.E.M. (*n* = 3). Different letters placed above columns represent significant differences between treatments (*p* < 0.05).

The activation of YAP/TAZ has been reported in many animal models of fibrosis-related kidney injury. We then used immunohistochemical methods to visualize protein expression of YAP. Conversely, administration of CCl4 resulted in an increase in YAP protein expression levels. However, treatment with IaC restored the YAP protein expression (Figure 6A). Our study demonstrated that CCl4 exposure led to an up-regulation of mRNA levels of *YAP* and *TAZ* in renal tissue. Conversely, treatment with IaC significantly down-regulated the mRNA expression of these genes. Western blot analysis further corroborated these findings, showing that CCl4 treatment enhanced the protein expression of YAP and TAZ in renal tissue compared to the control group. In contrast, IaC administration effectively reduced the expression of YAP and TAZ proteins in renal tissue when compared to CCl4 stimulation (Figure 6C–F). This evidence suggests that IaC may have improved renal function and reversed fibrosis by inhibiting the Hippo–YAP/TAZ pathway.

### 3.9. Inhibition of SESN2, YAP and MAPK Activation Partially Reversed the Effects of IaC in HK-2 Cells

**Figure 7:**
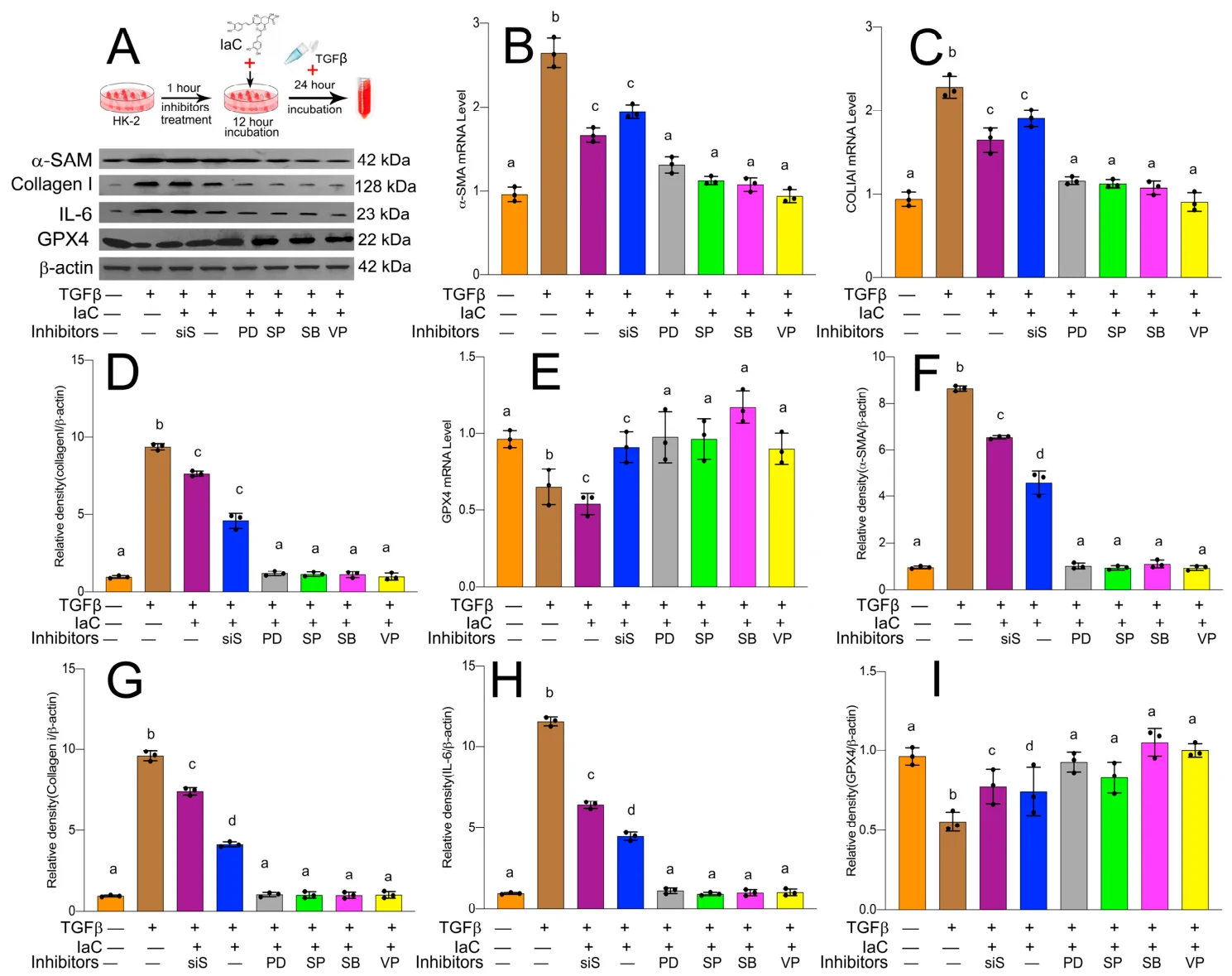
The inhibition of SESN2, GPX4, and YAP activation partially modulated the effects of isochlorogenic acid C (IaC) in HK-2 cells. (**A**) α-SMA, collagen I, IL-6, and GPX4 detected by Western blot; (**B**) *α-SMA* mRNA levels; (**C**) *COL1A1* mRNA levels; (**D**) *IL-6* mRNA levels; (**E**) *GPX4* mRNA levels; (**F**) the quantitative analysis of the α-SMA protein bands; (**G**) the quantitative analysis of the collagen I protein bands; (**H**) the quantitative analysis of the IL-6 protein bands; (**I**) the quantitative analysis of the GPX4 protein bands. β-actin was probed as the internal control in relative density analysis. siS: siSESN2; VP: YAP inhibitor Verteporfin; SP: JNK inhibitor SP600125; SB: p38 inhibitor SB203580; PD: ERK inhibitor PD98059. The vehicle control is set as 1.0. Data are expressed as mean ± S.E.M. (*n* = 3 for histological/molecular analyses). Different letters placed above columns represent significant differences between treatments (*p* < 0.05).

The SESN2/MAPK and YAP signaling pathways play a role in regulating ferroptosis, inflammation and fibrosis. To assess the effect of SESN2, YAP, and MAPK inhibition on inflammation and fibrosis marker expression in the TGFβ cell model, HK-2 cells were stimulated with TGFβ and treated with IaC. The results indicate that, in comparison to the TGF-β control group, IaC mitigated the increase in TGF-β-induced inflammation, fibrosis, and markers associated with ferroptosis (Figure 7). Further, to validate the role of SESN2, YAP, and MAPKs in kidney fibrosis, we performed a confirmation experiment using inhibitors of YAP (Verteporfin), JNK (SP600125 (SP)), p38 (SB203580), and ERK (PD98059) in HK-2 cells. Treatment with the inhibitors of MAPKs and YAP significantly enhanced the effects of IaC in TGFβ-stimulated HK-2 cells. Sestrin2 silencing reversed the effects of IaC in TGFβ-stimulated HK-2 cells.

### 3.10. Docking Results

To determine the potential mechanism of action of IaC, the docking analysis was performed for IaC at the active sites of the SESN2, GPX4 and YAP proteins in order to correlate with the in vivo findings in this study. AutoDock Vina 1.2x scores of −8.142 kcal/mol for SESN2, −7.687 kcal/mol for GPX4, and −8.629 kcal/mol for YAP indicated a stabilized binding of IaC to these targets.

**Figure 8:**
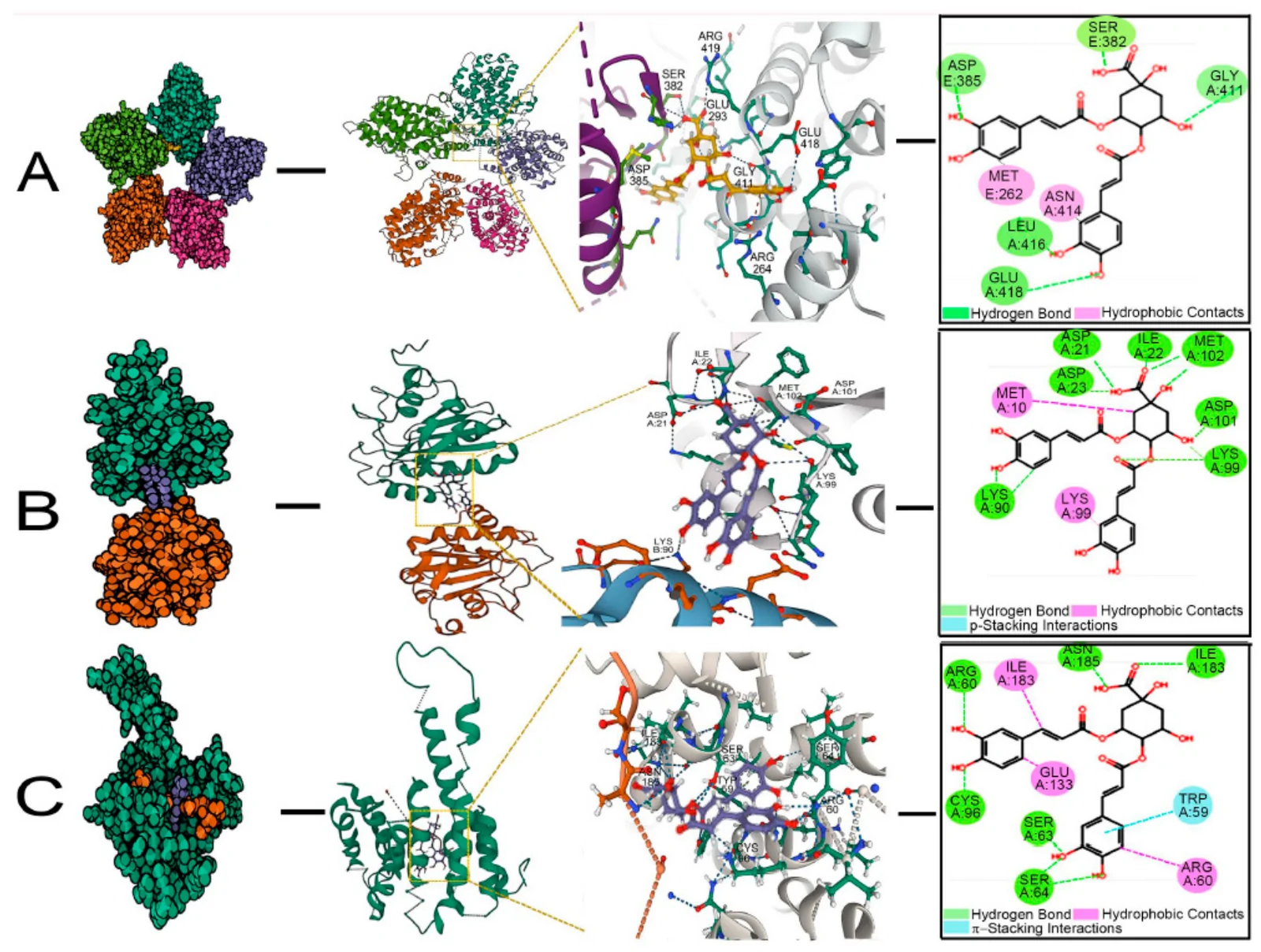
**Molecular docking of Isochlorogenic acid C (IaC) against target proteins SESN2, GPX4, and YAP.** (**A**) SESN2; **(B**) GPX4; (**C**) YAP.

On docking IaC with SESN2 (6N0M), IaC forms four hydrogen bonds with SESN2 key residues in GLY411A, GLU418A, LEU416A, and SER385E. Moreover, IaC is surrounded by the hydrophobic pocket of SESN2-IaC domain, where it is surrounded by aromatic amino acid residues (like ASN414A, and MET 262E) (Figure 8A). On docking IaC with GPX4 (9RF1), IaC forms four hydrogen bonds with GPX4 key residues in ASP 21A, ASP 23A, ILE22A, MET 102A, ASP101A, LYS 99A, and LYS90B. Moreover, IaC is surrounded by the hydrophobic pocket of the GPX4-IaC domain, where it is surrounded by aromatic amino acid residues (like LYS99A, and MET 102A) (Figure 8B). On docking IaC with YAP (6G6X), IaC forms six hydrogen bonds with YAP key residues in ILE 183A, ASN 185A, ARG 60A, CYS 96A, SER 63A and SER64A. IaC is surrounded by aromatic amino acid residues and forms hydrophobic interactions with amino acids (like GLU 133A, ILE 183A and ARG 60A). Furthermore, IaC engages Pi-Pi stacking interactions with TRP 59A (Figure 8C).

## 4. Discussion

Nephros fibrosis is the last stage of chronic nephropathy, during which a lot of extracellular matrix (ECM) is produced in the interstitial tubules [1,27]. CCl4 can cause fibrosis of the liver and kidneys as a result of inflammation [21,28]. Previous studies have demonstrated that IaC-rich extracts from stevia residue possess renoprotective properties by mitigating inflammation and fibrosis [29]. In the present study, we elucidate the potential therapeutic mechanisms of IaC in combating renal fibrosis. This was achieved through the use of TGFβ-induced stimulation in in vitro models and the induction of kidney fibrosis via CCl4 in a murine model. Our findings demonstrate that IaC effectively reduces TGFβ-induced collagen accumulation and down-regulates the expression of fibrotic markers in HK-2 cells. Moreover, the in vivo study indicated that IaC significantly alleviated CCl4-induced increases in oxidative stress, ferroptosis, inflammation, and fibrosis. According to this study, CCl4 increased the levels of serum creatinine, urea, and uric acid, indicating kidney damage. CCl4 also increased inflammatory cell infiltration and histological score of kidney damage (Figure 1). Interestingly, IaC alleviated kidney injury caused by CCl4.

In examining the underlying mechanisms, IaC not only reestablished oxidative stress equilibrium but also exhibited multi-target effects in the modulation of ferroptosis and inflammation, thereby supporting its considerable anti-fibrotic potential. This study provided robust evidence that IaC exerted significant renoprotective effects in a murine model of CCl4-induced renal injury. The findings indicated that CCl4 enhanced fibrotic activation and facilitated the overexpression of α-SMA and collagen I, which indicates the production of ECM and fibrosis [2,21]. Subsequently, these results were further confirmed by histological analysis (Figure 2). Previous studies showed that supplementation with isochlorogenic acid A and isochlorogenic acid B presented the anti-fibrosis effect in livers [30,31]. In this study, treatment with IaC decreased fibrosis area and myofibroblast activations in renal tissue, indicating its protective characteristics against kidney damage.

Oxidative stress is one of the main reasons for inflammation and fibrosis [30,32]. CCl4 could contribute to renal inflammation and fibrosis by promoting oxidative stress [2,21]. Furthermore, IaC has demonstrated significant antioxidative properties in mouse embryonic fibroblast cells [25]. As an antioxidant, it demonstrates stronger properties compared to the other two chlorogenic acid derivatives, isochlorogenic acids A and B. Isochlorogenic acid C has been shown to prevent EV71 infection by modulating the glutathione antioxidant system [33]. Additionally, IaC enhances the antioxidant effect in asthma-related lung damage by up-regulating the Nrf2 antioxidant system [34]. Results indicate that CCl4 increased MDA levels and decreased the activities of GSH, SOD, and GPx, displaying that it induced oxidative stress in kidneys. IaC supplementation could suppress oxidative stress in kidneys by decreasing ROS and restoring antioxidant enzyme activities [25]. Similarly, we found that SOD, and GPx activity increased along with MDA level declines in IaC treated mice compared to the CCl4 group (Table 1), implying that IaC treatment alleviated CCl4-induced kidney damage by ameliorating oxidative stress.

Ferroptosis represents a novel form of programmed cell death characterized by iron-dependent lipid peroxide accumulation and disruptions in lipid repair mechanisms, contributing to the progression of fibrotic diseases [35]. Increasing evidence indicates that ferroptosis plays a crucial role in the pathological mechanisms underlying CCl4-induced liver fibrosis [36,37,38]. Notably, IaC has demonstrated significant ferroptosis-inhibitory activity in BEAS-2B cells [39]. Following exposure to CCl4, an elevation in ferrous ions (Fe^2+^) was observed in the renal tissue. This accumulation of iron is a critical initiating factor in iron toxicity, as the surplus of unstable iron facilitates the Fenton reaction, which directly results in lipid peroxidation [36,40]. CCl4 also caused abnormal expression profiles of liver ferroptosis-related factors, including iron, GSH, GPX4, and SLC7A11 [37,38]. CCl4 exposure induced a coordinated dysregulation of ferroptosis-related proteins in kidneys, characterized by the suppression of GPX4 and SLC7A11 [41,42]. This result showed that CCl4 increased the Fe^2+^ level and decreased the expression of GSH, GPX4, and SLC7A11 (Figure 3). The simultaneous down-regulation of GSH, GPX4, and SLC7A11 significantly compromises the cellular capacity to counteract iron-induced lipid peroxidation, thereby delineating distinct molecular features of ferroptosis. Pretreatment with IaC markedly reinstated the expression levels of markers associated with oxidative stress and ferroptosis. These collective findings indicate that IaC demonstrates potential efficacy in mitigating markers associated with ferroptosis.

Many studies confirmed that renal fibrosis was correlated well with inflammation [2,28]. CCl4 induction could accelerate inflammation in kidney by stimulating the production of pro-inflammatory mediators [2,3,21]. CCl4 exposure has been shown to enhance the expression of TGFβ, a recognized pivotal mediator in the development of renal tubular fibrosis [2,21]. Additionally, TGFβ plays a role in promoting the secretion of pro-inflammatory factors within renal cells [43]. IaC-rich extracts of stevia residue remarkably modulate these inflammation-related genes by inhibiting the NF-κB signaling pathway [29]. The NF-κB pathway is activated in response to various stimuli, including pro-inflammatory cytokines and oxidative stress, and mediates several key processes that contribute to fibrosis progression [44]. The activation of the NF-κB signaling pathway facilitates the differentiation of fibroblasts into myofibroblasts, consequently intensifying the severity of fibrotic diseases. Additionally, the NF-κB signaling pathway interacts with the TGF-β/Smad3 pathway, further promoting the progression of tissue fibrosis [44,45]. Conversely, IaC treatment has been demonstrated to modulate reverse cholesterol transport and attenuate inflammation both in vivo and in vitro by inhibiting the NF-κB signaling pathway [24]. Furthermore, IaC has been reported to alter the gut microbiota composition, influence immune cell function, and mitigate airway inflammation in a murine model of asthma [34]. Indeed, CCl4 also stimulated the activations of NF-κB, TNF-α and IL-6 in mouse kidneys, and IaC suppressed the NF-κB activation and inflammation by decreasing the release of pro-inflammatory mediators (Figure 4). Based on these findings, IaC improved renal fibrosis, and inflammation.

The SESN2 and MAPK signaling pathways are integral to the regulation of inflammatory and fibrotic mechanisms implicated in renal injury [46,47,48,49]. The administration of CCl4 has been demonstrated to induce hepatic inflammation and fibrosis by down-regulating SESN2 and up-regulating MAPK phosphorylation [16,17,50]. Further studies have shown that SESN2 inhibition reduces MAPK phosphorylation, indicating that SESN2 may regulate MAPK-related signaling pathways involved in ferroptosis, inflammation, and apoptosis [5,6]. The anti-oxidative protein SESN2 exhibits the ability to suppress inflammation, oxidative stress, and cell death [50,51]. It is reported that Isochlorogenic acid A inhibited itching and inflammation by regulating NF-kB and MAPK pathways [52]. Isochlorogenic acid A prevented rheumatoid arthritis by regulating the MAPK pathway [53]. The application of MAPK inhibitors abrogated the antioxidant effect of IaC in fibroblast cells [25]. IaC has been reported to mitigate oxidative stress and inflammation vis the MAPK/NF-κB pathway [34]. This study further examined the SESN2/MAPK pathway to determine the potential mechanism by which IaC inhibits inflammation and fibrosis caused by CCl4 (Figure 5). The results showed that the expression of p-ERK1/2, p-JNK1/2 and p-p38 markedly escalated the CCl4 group when compared to the control. After IaC treatment, these protein expression dramatically reduced. IaC also activated the SESN2 pathway to improve renal inflammation and fibrosis caused by CCl4.

The activation of the Hippo signaling pathway plays a pivotal role in the pathogenesis of fibrotic diseases [54,55]. Additionally, the YAP/TAZ signaling pathway facilitates the production of pro-inflammatory mediators across various organs and tissues [55,56,57]. Previous research has demonstrated that the induction of CCl4 can lead to hepatic inflammation and fibrosis through the activation of YAP α-SMA, collagen I, TGF-β, TNF-α, and IL-6 [18,20,55]. YAP plays a regulatory role in modulating the downstream fibrotic and inflammatory target ligand TGF. Inhibition of YAP has been associated with a reduction in kidney inflammation and fibrosis [55,58]. SESN2 signaling is capable of inducing phosphorylation at multiple sites on the YAP protein, resulting in the inhibition of its activity and promoting its degradation via the proteasome. This process consequently leads to the suppression of myofibroblast formation [15,55,59]. SESN2 overexpression alleviated mesangial cell proliferation, ECM deposition and fibrosis in diabetic nephropathy by regulating Hippo–YAP signaling pathway [47]. Chlorogenic acid has been reported to suppress vein graft restenosis, inflammation, and fibrosis by modulating the Hippo–YAP axis [60]. The current findings revealed that IaC could down-regulate the expression of YAP to inhibit fibrosis and the release of inflammatory cytokines, thus decrease CCl4-induced kidney injury (Figure 6).

Moreover, this study confirmed that IaC treatment suppressed TGFβ-induced inflammation and fibrosis in HK-2 cells (Figure 7), thus alleviating kidney damage through inhibition of inflammation. However, treatment with the inhibitors of MAPKs and YAP significantly enhanced the effects of IaC in TGFβ-stimulated HK-2 cells. Sestrin2 silencing reversed the effects of IaC in TGFβ-stimulated HK-2 cells. Overall, these findings demonstrated that IaC treatment dramatically lowered crucial indicators of inflammation and fibrosis by the SESN2/MAPK and Hippo pathways.

To elucidate the interactions between IaC and SESN2, GPX4, YAP, we constructed models of the 3D binding conformations of IaC within the binding pockets of SESN2 (PDB ID: 6N0M), GPX4 (PDB ID: 9RF1), and YAP (PDB ID: 6G6X), followed by docking analysis. The formation of hydrogen bonds, hydrophobic contacts, and Pi-Pi stacking interactions is critical for internal stabilization and molecular recognition [61,62]. The docking prediction indicates a correlation between IaC and SESN2, GPX4, and YAP (Figure 8). Computational docking analysis shows that IaC can interact with the binding sites of SESN2 and GPX4 with strong affinity. IaC can also establish hydrogen bonds and Pi-Pi interactions with YAP. This interaction corroborates the anticipated compatibility or binding affinity of IaC with these proteins.

## 5. Conclusions

**Figure 9:**
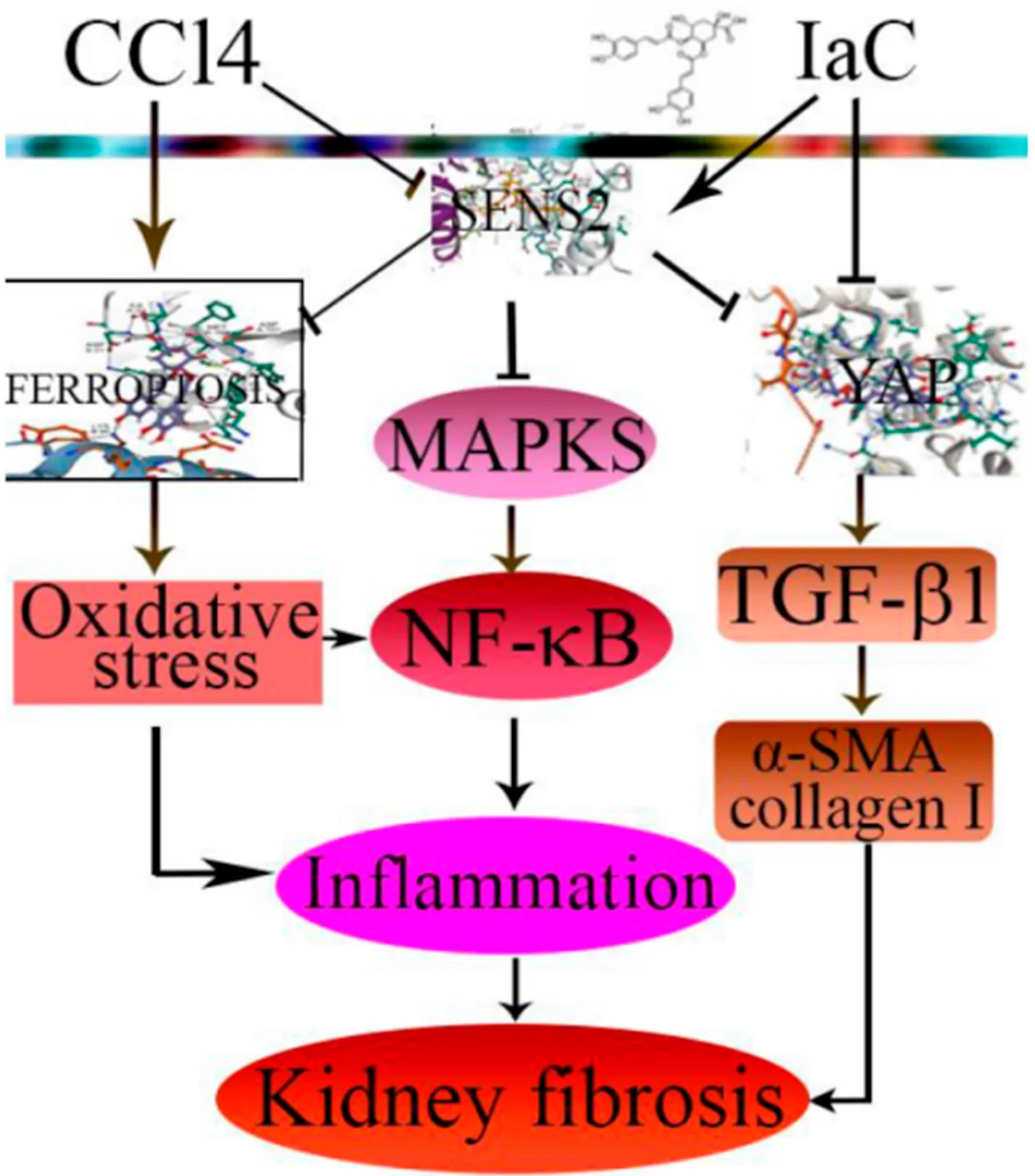
Schematic diagram shows the possible protective effects of Isochlorogenic acid C (IaC) in CCl4-induced kidney injury. The ↑ indicates activation or induction, and ┣ indicates inhibition or blockade.

Our study demonstrates that IaC can dramatically ameliorate CCl4-induced oxidative stress, ferroptosis, inflammation, and fibrosis via the Hippo pathway and the SESN2/MAPKs and YAP pathways (Figure 9). These findings provide a molecular basis for the potential anti-fibrotic activity of IaC and support its further investigation as a therapeutic candidate. This study is subject to certain limitations, primarily due to its reliance on a single animal model experiment and a restricted sample size. Further validation and analysis of additional mechanisms are necessary.

## Figures and Tables

**Table 1:** Primers for quantitative PCR.

Primer Name	Forward (5′-3′)	Reverse (5′-3′)
Gene (Mouse)		
*α-SMA*	TGCTGACAGAGGCACCACTGAA	CAGTTGTACGTCCAGAGGCATAG
*COL1A1*	GACCAAAAGGTGATGCTGGACAG	CAAGACCTCGTGCTCCAGTTAG
*IL-6*	TACCACTTCACAAGTCGGAGGC	CTGCAAGTGCATCATCGTTGTTC
*TNF-α*	CCCTCACACTCAGATCATCTTCT	GCTACGACGTGGGCTACAG
*TGFß1*	ATACGCCTGAGTGGCTGTCT	CGTCGAGTTTGTTATCTTTGCTGT
*SLC7A11*	TGGGTGGAACTGCTCGTAAT	AGGATGTAGCGTCCAAATGC
*GPX4*	CATGCCCGATATGCTGAGTGTGG	TAGCACGGCAGGTCCTTCTCTATC
*SESN2*	ATACCATCGCCATGCACAGT	AGCTGGTTCACCTCCCCATA
*YAP*	TGGGAGATGGCCAAGACATC	CATGTTGTTGTCTGATCGTTGTGA
*TAZ*	CAGAAACTGCGGCTTCAGAG	GTTTCCATGGGGAGCTGTC
*β-Actin*	AGTGTGACGTTGACATCCGT	TGCTAGGAGCCAGAGCAGTA
Gene (Human)		
*α-SMA*	CACCATCACCCCCTGATGTC	ACTGCCTTGGTGTGTGACAA
*COL1A1*	TGGTCTGCAAGGAATGCCTGGA	TCTTTCCCTGGGACACCATCAG
*IL-6*	AGACAGCCACTCACCTCTTCAG	TTCTGCCAGTGCCTCTTTGCTG
*GPX4*	GGCTTCGTGTGCATCGTCACC	TTCACCACGCAGCCGTTCTTG
*β-Actin*	GGGAAATCGTGCGTGACATT	GGAACCGCTCATTGCCAAT

**Table 2:** Effect of IaC on CCl4-induced oxidative stress in kidneys of mice.

	MDA (mol/mg Protein)	SOD (U/mg Protein)	GPx (U/mg Protein)	GSH (μmol/g Protein)
Control	0.31 ± 0.05 ^a^	246.39 ± 14.45 ^a^	111.78 ± 14.40 ^a^	81.74 ± 9.03 ^a^
CCl4	0.49 ± 0.13 ^b^	139.10 ± 7.23 ^b^	82.06 ± 6.56 ^b^	31.36 ± 7.29 ^b^
CCl4 + IaC (10 mg/kg)	0.42 ± 0.04 ^c^	196.34 ± 16.02 ^c^	98.45 ± 4.39 ^c^	50.13 ± 7.32 ^c^
CCl4 + IaC (20 mg/kg)	0.36 ± 0.04 ^d^	220.14 ± 19.98 ^d^	108.73 ± 8.17 ^d^	65.97 ± 6.44 ^d^
IaC (20 mg/kg)	0.29 ± 0.05 ^a^	247.26 ± 18.33 ^a^	111.98 ± 6.71 ^a^	81.90 ± 6.62 ^a^

Data are expressed as mean ± S.E.M. (*n* = 7 for biochemical analyses). One-way ANOVA was used for comparisons of multiple group means followed by post hoc testing. Different letters placed above the data represent significant differences between treatments (*p* < 0.05).

## Data Availability

The original contributions presented in this study are included in the article. Further inquiries can be directed to the corresponding authors.

## Abbreviations

CAT	catalase
ERK	extracellular signal-regulated kinase
GPx	glutathione peroxidase
α-SMA	α-smooth muscle actin
SOD	superoxide dismutase
IaC	isochlorogenic acid C
IL-6	interleukin-6
JNK	N-terminal kinase
NF-κB	nuclear factor-κB
MAPK	mitogen-activated protein kinase
TAZ	transcriptional coactivator with PDZ-binding motif
TGF-β	transforming growth factor β
TNF-α	tumor necrosis factor-alpha
YAP	Yes-associated protein
